# UV Absorption Spectra of TAMRA and TAMRA Labeled Peptides: A Combined Density Functional Theory and Classical Molecular Dynamics Study

**DOI:** 10.1002/jcc.70096

**Published:** 2025-03-31

**Authors:** Mercedes Kukulka, Barbara Pem, Katarina Vazdar, Lukasz Cwiklik, Mario Vazdar

**Affiliations:** ^1^ Faculty of Chemistry Jagiellonian University Krakow Poland; ^2^ Division for Organic Chemistry and Biochemistry Ruđer Bošković Institute Zagreb Croatia; ^3^ J. Heyrovský Institute of Physical Chemistry of the Czech Academy of Sciences Prague Czech Republic; ^4^ Department of Mathematics Informatics and Cybernetics, University of Chemistry and Technology Prague Czech Republic

**Keywords:** fluorescent probes, molecular dynamics simulations, time‐dependent density functional theory, UV absorption spectra

## Abstract

This study explores the structural and electronic factors affecting the absorption spectra of 5‐carboxy‐tetramethylrhodamine (TAMRA) in water, a widely used fluorophore in imaging and molecular labeling in biophysical studies. Through molecular dynamics (MD) simulations and density functional theory (DFT) calculations, we examine TAMRA UV absorption spectra together with TAMRA‐labeled peptides (Arg_9_, Arg_4_, Lys_9_). We found that DFT calculations with different functionals underestimate TAMRA maximum UV absorption peak by ~100 nm, resulting in the maximum at ca. 450 nm instead of the experimental value of ca. 550 nm. However, incorporating MD simulation snapshots of TAMRA in water, the UV maximum peak shifts and is in close agreement with the experimental results due to the rotation of TAMRA N(CH_3_)_2_ groups, effectively captured in MD simulations. The method is used to estimate the UV absorption spectra of TAMRA‐labeled peptides, matching experimental values.

## Graphical abstract

This study examines the UV absorption spectra of TAMRA using TD‐DFT calculations and MD simulations. The B3LYP functional and 6‐31+G(d) basis set were optimal for modeling TAMRA's electronic transitions. MD simulations revealed that the dimethylamine group's rotational flexibility significantly impacts the absorption peak, and TD‐DFT calculations using MD snapshots result in good agreement with experimental data. Additionally, TAMRA's conjugation to peptides did not alter its main absorption features.

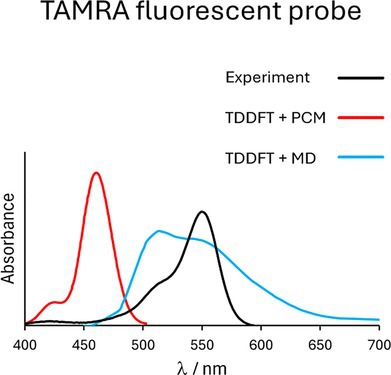

## 1 Introduction

Understanding the behavior of fluorescent probes at the molecular level is crucial for their effective application in biophysical research on model biological systems and living cells [1, 2]. 5‐Carboxytetramethylrhodamine (TAMRA), a widely used fluorophore, plays a significant role in fluorescence imaging, molecular labeling, and biophysical assays due to its strong UV absorption and bright fluorescence emission [3]. To maximize the utility of TAMRA in model biological systems, it is useful to explore the structural and electronic properties that govern its absorption spectra. Molecular dynamics (MD) simulations, combined with density functional theory (DFT) calculations, provide a powerful approach for investigating these properties at an atomic level, enabling insights into the relationship between TAMRA structure and its UV absorption behavior. So far, a detailed structural and computational analysis of TAMRA in correlation with its UV absorption spectra has not been published, although other probes, such as PRODAN [4] and BODIPY [5], have been studied in the literature.

TAMRA and TAMRA‐labeled molecule UV absorption spectra are primarily determined by their electronic transitions, which are influenced by the fluorophore conformation and interactions with its environment. TAMRA is often conjugated in biological systems to biomolecules such as proteins or nucleic acids [6, 7], where solvent effects, binding interactions, and local molecular conformations may alter its structural dynamics. MD simulations allow for detailed modeling of TAMRA structural flexibility in different environments, while computational methods such as time‐dependent density functional theory (TD‐DFT) can predict the absorption spectra based on these structures. Such insights are essential for optimizing TAMRA use as a probe in various biological contexts, from fluorescence resonance energy transfer (FRET) [8] to live‐cell imaging [9]. Moreover, understanding how TAMRA absorption spectrum shifts under different conditions can aid in designing improved fluorophores with enhanced performance in complex biological systems.

This study combines DFT calculations and MD simulations, comparing them with experimental UV–vis spectral analysis to elucidate the structural factors that affect TAMRA and TAMRA‐labeled peptides (Arg_9_, Arg_4_, and Lys_9_) UV absorption characteristics. First, we use TD‐DFT calculations of TAMRA in the PCM continuum water model [10, 11] to establish the optimal DFT functional for calculating absorption spectra. In the next step, instead of PCM geometries, we use snapshots from MD simulations to compare the results with the experimental UV–vis spectra and examine in detail the differences between the two approaches.

## 2 Methods

### 2.1 Density Functional Theory Calculations

(TD‐)DFT calculations of TAMRA and TAMRA‐labeled peptides were performed using the Gaussian 16 package, revision A.03 [12]. The optimized geometries in the ground state were obtained using a gradient (BP86 [13, 14], BLYP [13, 15]), meta‐gradient (TPSS [16]), meta‐hybrid (M06‐2X [17], TPSSh [16, 18]), and hybrid (PBE0 [19], B3LYP [20], CAM‐B3LYP [21], wB97XD [22]) functionals. The DFT approach with Grimme's D3 dispersion scheme and Becke‐Johnson BJ damping (referred to as DFT‐D3BJ; D3 for the M06‐2X functional as empirical parameters for a BJ‐damped D3 correction are not available) was also employed [23, 24]. The split‐valence double‐ζ and triple‐ζ basis sets, including one set of polarization functions for all atoms or only for non‐hydrogen atoms as well as diffuse functions, were used: 6‐31+G(d), 6‐311+G(d,p), 6‐311G(d,p) [25, 26], and aug‐cc‐pVDZ [27, 28, 29]. Subsequent TD‐DFT calculations of UV–vis absorption spectra were performed at the same level of theory. The absorption calculations covered 3, 5, and 20 (for confirmation) lowest‐energy singlet excited states, and the corresponding UV–vis spectra were simulated as the sums of Gaussian functions centered at the vertical excitation energies and scaled using the computed oscillator strengths with the half‐width at half‐height factor set to 0.1 eV. Solvent effects were included in all calculations using the polarizable continuum model (PCM) for water (*ε* = 78.3553) [11, 30]. To better assess the accuracy of the DFT results regarding conformational preferences of TAMRA, geometry optimization calculations were carried out at the Møller–Plesset second‐order perturbation theory (MP2) level [31], employing the resolution‐of‐identity (RI) approximation for MP2 integrals. The cc‐pVTZ basis set [27] and the corresponding cc‐pVTZ/C RI‐C auxiliary basis set [32] were used alongside the conductor‐like PCM model (CPCM) for water [11]. MP2 calculations were performed using the ORCA software (version 5.0.4) [33].

### 2.2 Molecular Dynamics Simulations

Simulations of TAMRA and TAMRA‐labeled peptides in water were conducted using classical MD simulations. The parameters for the TAMRA residue were obtained from CGenFF [34]. The charges were adjusted to ensure the symmetric distribution throughout the tricyclic segment due to its resonant properties, as seen in Refs [35, 36] and the total charge of TAMRA was −1. The amino acid parameters were taken from the CHARMM36m force field [37], and the TIP3P model was used for water [38]. The simulation boxes contained one molecule of either TAMRA‐Arg_4_, TAMRA‐Arg_9_, TAMRA‐Lys_9_, or TAMRA alone, solvated with 4105, 9668, 9855, or 1925 waters, respectively. Na^+^ or Cl^−^ ions were added to ensure neutrality. Following minimization, the systems were heated to 298 K for 200 ps in the NVT ensemble. The production was run for 20 ns in the NpT ensemble with the Nosé–Hoover thermostat (coupling constant of 1 ps), and the pressure was kept at 1 bar with the Parrinello–Rahman barostat (coupling constant of 2 ps). Long‐range electrostatic interactions were handled by the particle mesh Ewald (PME) method. The interaction cutoff was 1.2 nm, with the switching function employed after 1 nm. LINCS was used to constrain the bonds involving hydrogen, allowing for the 2 fs time step. 3D periodic boundary conditions were used throughout. All simulations were conducted in GROMACS 2020 [39].

20 ps of MD simulations of a simplified TAMRA model, 3‐(dimethylamino)phenolate with charge −1, and solvated with 50 water molecules were performed using Born–Oppenheimer molecular dynamics (BOMD) at the B3LYP/6‐31G(d) level of theory and a 1 fs time step. Spherical boundary conditions were applied, keeping constant density in the system of 1 kg dm^−3^ and a force constant of 1.0 kcal mol^−1^ Å^−2^ needed to prevent water evaporation. The temperature was maintained at 300 K using a velocity‐rescale thermostat [40] with a rescaling frequency of every 10 fs. The BOMD simulations were performed using Terachem 1.9.3 software [41, 42].

### 2.3 Experiments

TAMRA was purchased from Sigma‐Aldrich. 5(6)‐Carboxytetramethylrhodamine‐(Lys)9‐OH (or TAMRA‐Lys_9_), 5(6)‐carboxytetramethylrhodamine (Arg)4‐OH (or TAMRA‐Arg_4_) and 5(6)‐carboxytetramethylrhodamine‐(Arg)9‐OH (TAMRA‐Arg_9_) were synthesized according to the published protocol.

Absorption spectra were recorded on a Shimadzu UV2600 spectrometer at room temperature. The selected compounds were prepared as 5 μM solutions in phosphate‐buffered saline (PBS). PBS was prepared by dissolving 1x PBS tablets (Merck, Darmstadt, Germany) in Milli‐Q (MQ) water, filtered, at pH 7.4. All the spectra were measured in 1 mL quartz cuvettes (path length 10 mm) and collected in 1 nm steps.

## 3 Results and Discussion

### 3.1 Optimized Geometry in the Ground State

To establish a computationally efficient yet reliable protocol for describing the absorption properties of TAMRA‐labeled peptides, initial test calculations were performed on the TAMRA dye. The key aspects addressed include (i) the choice of DFT functional, (ii) the size of the basis set and the necessity of diffuse functions, (iii) the need for dispersion correction, and (iv) the number of excited states considered.

**FIGURE 1:**
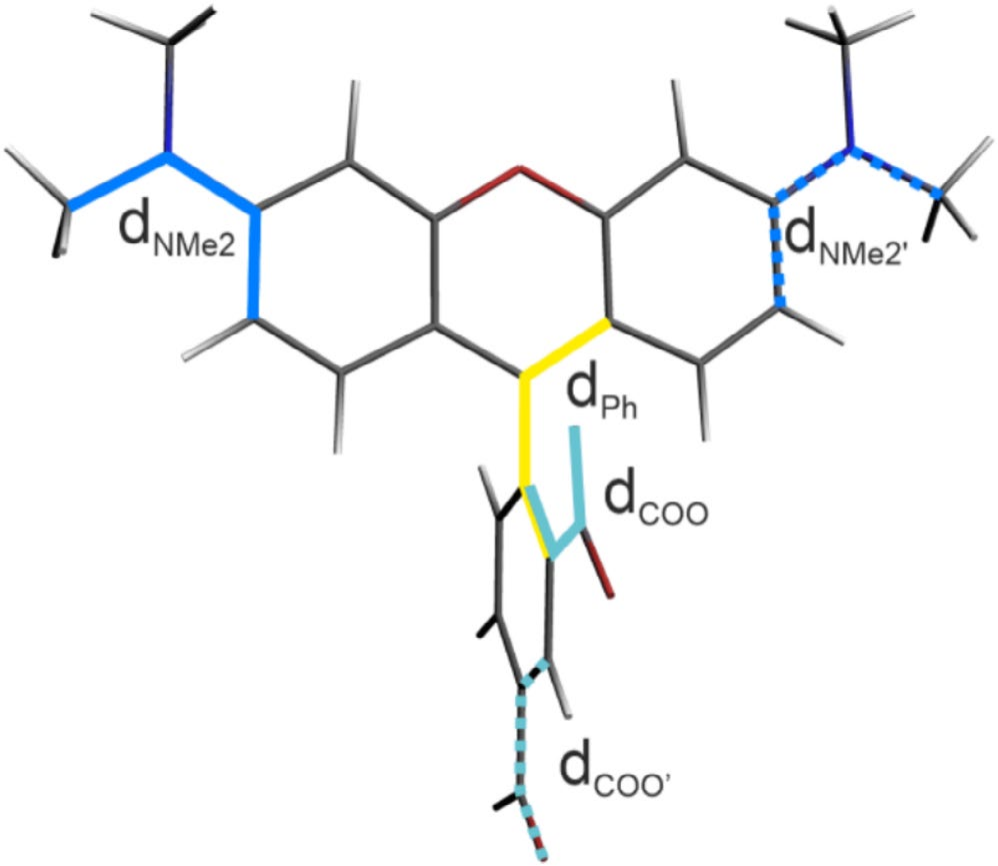
Optimized ground‐state structure of TAMRA (B3LYP/6‐31+G(d)/PCM(H_2_O)) with the relevant dihedral angles marked (see Table 1 for the values).

The DFT geometry optimization calculations for TAMRA in the ground state, considering different orientations of the dicarboxyphenyl and dimethylamine groups with respect to the xanthene fragment, resulted in a similar structure, regardless of the computational details used (DFT functional, basis set, dispersion correction), where the planar dicarboxyphenyl unit is nearly perpendicular to the planar 3‐*N*,3‐*N*,6‐*N*,6‐*N*‐tetramethyl‐9*H*‐xanthene‐3,6‐diamine moiety (Figure 1). The corresponding dihedral angle value varies from 91° to 99°. An exception is observed with the M06‐2X functional, where it is somewhat lower: 73° with D3 correction and 82° without. MP2 calculations confirmed conformational preferences. See Table 1 for the full set of data.

**TABLE 1:** Selected dihedral angle values (in °) for optimized structures of TAMRA using different DFT functionals.

Functional	*d*_Ph_	*d*_COO_	*d*_COO′_	*d*_NMe2_	*d*_NMe2′_
6‐31+G(d)/PCM(H_2_O)					
BP86	96.9	−0.3	−1.8	−0.1	−0.1
BLYP	96.2	−0.7	−2.4	0.5	−0.2
TPSS	96.7	−0.3	−1.7	−0.0	−0.1
B3LYP	91.5	3.6	−0.7	0.1	−0.3
PBE0	98.4	−2.6	1.6	0.0	−0.2
TPSSh	96.5	−0.8	−1.6	0.0	−0.2
M06‐2X	82.1	11.7	0.6	0.2	−0.3
CAM‐B3LYP	95.5	−1.7	−1.6	0.1	−0.3
wB97XD	91.2	3.9	−0.8	0.1	−0.2
D3(BJ)/6‐311+G(d,p)/PCM(H_2_O)					
BP86	97.6	−0.6	−2.0	0.1	−0.2
BLYP	97.9	−2.5	−3.1	0.3	−0.4
TPSS	97.2	−0.5	−1.7	0.1	−0.2
B3LYP	98.5	−3.5	1.6	0.2	−0.4
PBE0	98.7	−2.8	1.5	0.1	−0.2
TPSSh	99.3	−2.4	1.7	0.1	−0.2
M06‐2X	72.7	23.2	1.2	0.3	−0.4
CAM‐B3LYP	97.5	−3.2	1.6	0.2	−0.3
MP2/cc‐pVTZ/CPCM(H_2_O)					
	97.5	−3.2	1.6	0.2	−0.3

*Note:* For clarity, only results with the 6‐31+G(d) basis set (without dispersion correction) and for 6‐311+G(d,p) (with D3BJ correction, D3 for M06‐2X) are shown. For comparison, the results from MP2 are presented. See Figure 1 for the dihedral angle designation.

### 3.2 UV–Vis Spectra

**FIGURE 2:**
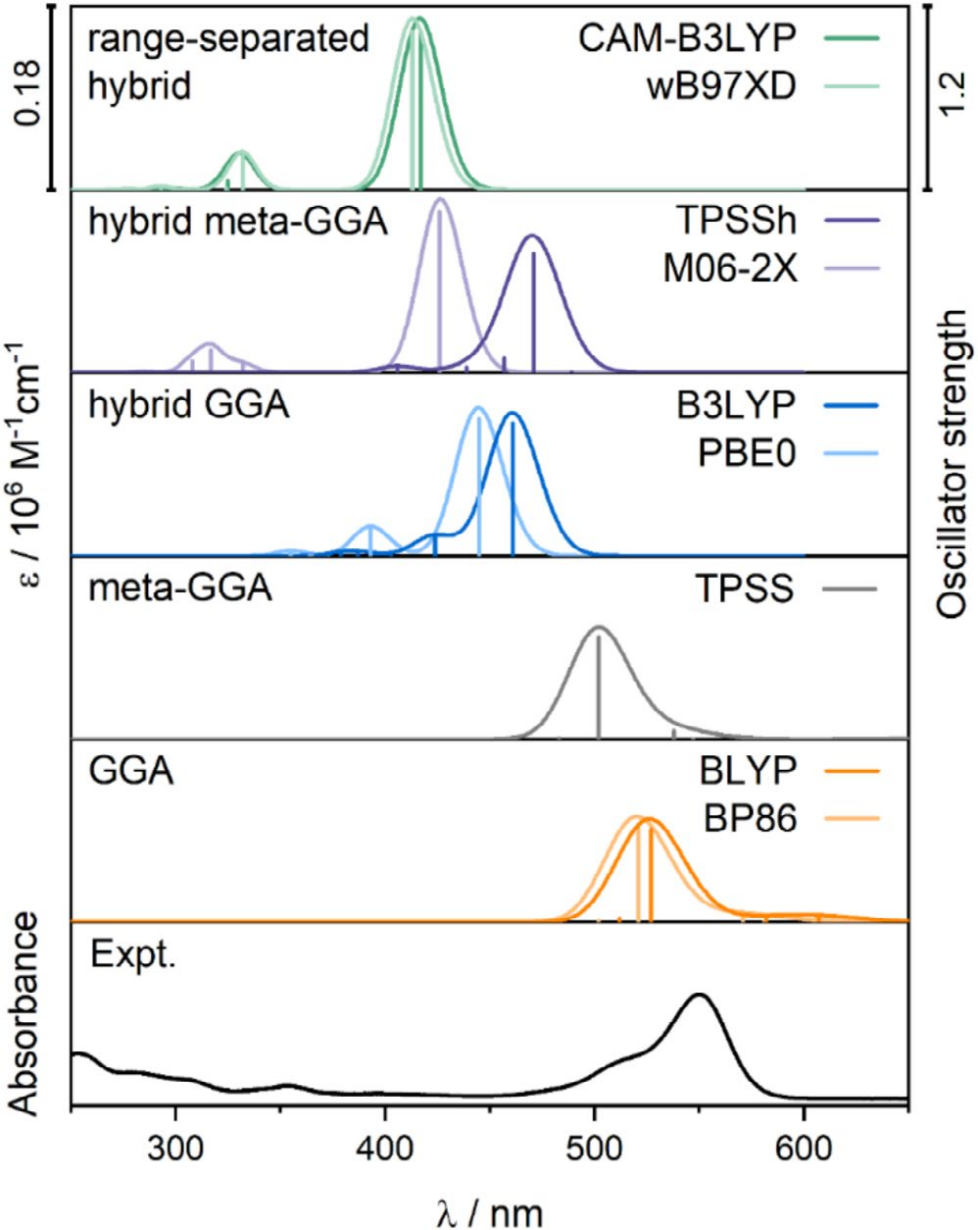
TD‐DFT simulated UV–vis spectra for TAMRA calculated at different functionals (6‐31+G(d)/PCM(H_2_O); five excited states) along with the experimental spectrum in PBS. Calculated excitation energies and oscillator strengths are represented as stick spectra.

As shown in Figure 2, in the low energy range, the experimental spectrum of the TAMRA dye exhibits a strong absorption band centered at 550 nm, followed by a less intense band around 510 nm, with non‐zero absorption intensity extending up to approximately 400 nm. The best reproduction of the spectral features in the low‐energy range was achieved with B3LYP, despite a 90 nm blue shift compared to the experimental spectrum (Figure 2). However, a hypsochromic shift of the lowest‐energy band is observed for all tested functionals, the most significant for the long‐range corrected functionals due to their range‐separated exact‐exchange contribution. Among the GGA and meta‐GGA functionals, the intense absorption band is closest to the experimental results; however, the first excitation with low oscillator strength occurs beyond 600 nm or even 700 nm in the case of GGA functionals (see Table 2).

**TABLE 2:** Selected excitations and corresponding occupied → unoccupied molecular orbital (MO)‐pair contributions (> 10%) for TAMRA.

Functional	Excit. no.	*E* (eV)	*λ* (nm)	*f*	Occ no.	Unocc. no.	(%)	Character
BP86	1	1.73	718	0.0055	113	114	99.9	CT
	2	2.13	581	0.0016	110	114	94.9	CT
	3	2.17	571	0.0508	111	114	73.0	CT
					109	114	18.1	pCT
	4	2.38	521	0.6351	112	114	95.2	*π*–π*
BLYP	1	1.70	730	0.0054	113	114	99.9	CT
	2	2.04	607	0.0340	111	114	75.3	CT
					110	114	15.1	CT
	3	2.13	582	0.0208	110	114	82.8	CT
	4	2.35	527	0.6099	112	114	91.2	*π*–π*
TPSS	1	1.88	659	0.0048	113	114	99.9	CT
	2	2.27	547	0.0016	110	114	98.7	CT
	3	2.30	538	0.0554	111	114	75.8	CT
					109	114	18.5	pCT
	4	2.47	502	0.6713	112	114	95.7	*π*–π*
B3LYP	1	2.69	461	0.8692	113	114	98.6	*π*–π*
	2	2.92	424	0.1259	112	114	95.1	pCT
PBE0	1	2.79	445	0.8993	113	114	98.5	*π*–π*
	2	3.15	393	0.1809	112	114	95.2	pCT
TPSSh	1	2.54	489	0.0028	112	114	99.6	*π*–π*
	2	2.63	471	0.7810	113	114	97.9	CT
M06‐2X	1	2.91	426	1.0537	113	114	97.1	*π*–π*
	2	3.74	332	0.0598	112	114	77.4	*π*–π*
	3	3.91	317	0.1513	111	114	73.1	pCT
CAM‐B3LYP	1	2.97	417	1.0384	113	114	96.3	*π*–π*
	2	3.74	332	0.1898	112	114	90.4	pCT
wB97XD	1	3.00	413	1.0368	113	114	95.1	*π*–π*
	2	3.73	332	0.2229	112	114	84.4	pCT

*Note:* DFT/6‐31+G(d)/PCM(H_2_O) calculations involving five lowest‐energy excited states.

**FIGURE 3:**
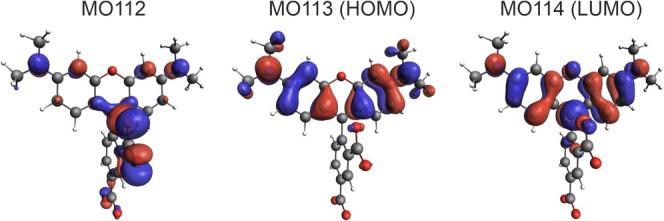
Isosurfaces (± 0.03 au) of MOs involved in the dominant transitions of TAMRA. B3LYP/6‐31+G(d)/PCM(H_2_O) calculations.

Table 2 indicates that, for B3LYP calculations, the lowest‐energy band in the absorption spectrum originates from Excitation 1, which can be assigned to a *π*–π* transition from the highest‐occupied molecular orbital (HOMO) to the lowest‐unoccupied molecular orbital (LUMO), localized within the 3‐*N*,3‐*N*,6‐*N*,6‐*N*‐tetramethyl‐9*H*‐xanthene‐3,6‐diamine fragment (see Figure 3). The second band is dominated by Excitation 2, which can be described as a HOMO‐1‐to‐LUMO transition, involving charge transfer (CT) from the carboxylate group to the xanthene moiety. The assignments are consistent for the other DFT functionals (Figure S1); namely, the most intense excitation exhibits a pure *π*–π* character, while the remaining excitations are characterized as either CT or partial CT (pCT).

**FIGURE 4:**
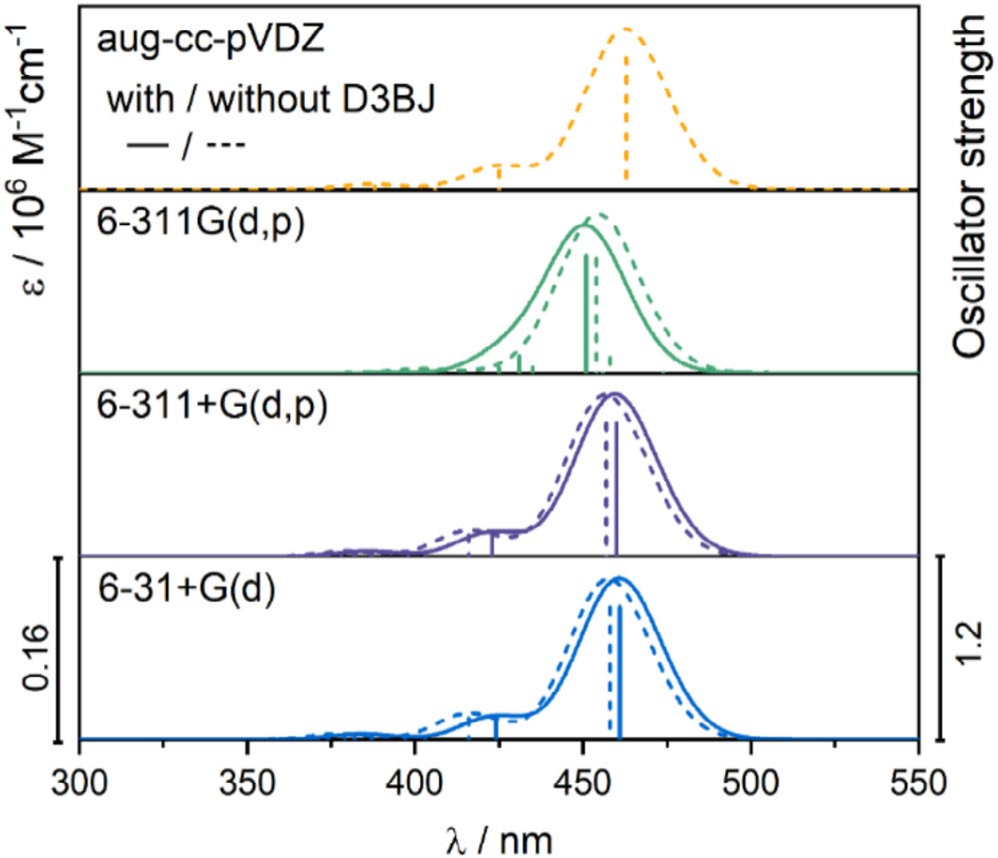
TD‐DFT simulated UV–vis spectra of TAMRA for the five lowest‐energy excited states, calculated using B3LYP with and without D3BJ dispersion correction, employing a continuum solvent model for water and different basis sets.

To investigate the sensitivity of the calculated absorption spectrum of the dye to the size of the basis set and the introduction of dispersion correction, additional test computations were performed using B3LYP with various basis sets and with/without dispersion correction. The spectra obtained with the split‐valence double‐ζ basis set, 6‐31+G(d), are nearly indistinguishable from those based on calculations using the larger triple‐ζ 6‐311+G(d,p) basis set as well as Dunning aug‐cc‐pVDZ (Figure 4). Similarly, applying the D3BJ dispersion correction during geometry optimization has only a minor impact on the spectrum, resulting in a blue shift of the lowest‐energy band by approximately 5 nm. As expected, diffuse functions are necessary, as simulations without them result in the two excitations being too close in energy, with the higher‐energy excitation having too low oscillator strength to be distinguished as separate bands in the spectrum (Figure 4, Table 3).

**TABLE 3:** Selected excitations and corresponding occupied → unoccupied MO‐pair contributions (> 10%) for TAMRA.

Basis set	Dispersion correction	Excit. no.	*E* (eV)	*λ* (nm)	*f*	Occ no.	Unocc no.	(%)
6‐31+G(d)	D3BJ	1	2.71	458	0.8659	113	114	98.6
		2	2.98	416	0.1413	112	114	94.6
6‐311+G(d,p)	—	1	2.70	460	0.8733	113	114	98.5
		2	2.93	423	0.1296	112	114	95.3
6‐311+G(d,p)	D3BJ	1	2.71	457	0.8705	113	114	98.5
		2	2.98	416	0.1392	112	114	95.1
6‐311G(d,p)	—	1	2.72	456	0.0013	112	114	99.4
		2	2.75	451	0.7715	113	114	96.3
		3	2.88	431	0.1131	111	114	65.0
						110	114	25.6
6‐311G(d,p)	D3BJ	1	2.62	474	0.0014	113	114	99.3
		2	2.71	458	0.1014	111	114	85.3
		3	2.73	454	0.7474	112	114	92.8
aug‐cc‐pVDZ	—	1	2.68	463	0.8668	113	114	98.5
		2	2.92	425	0.1295	112	114	95.3

*Note:* Calculations involving the five lowest‐energy excited states were performed using B3LYP with the PCM solvent model for water, both with and without dispersion correction, and employing different basis sets. For results from 6‐31+G(d) basis set without D3BJ, see Table 2.

**FIGURE 5:**
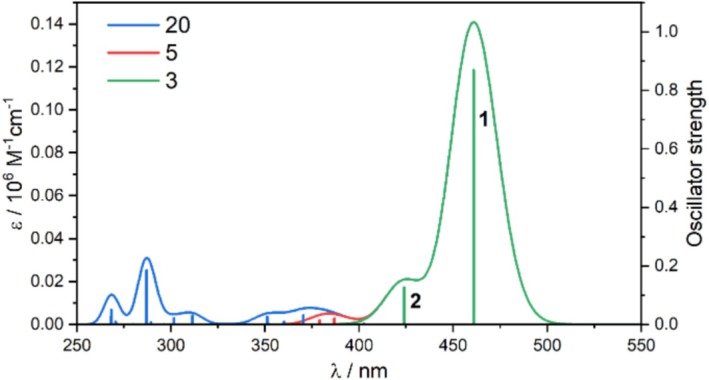
TD‐DFT (B3LYP/6‐31+G(d)/PCM(H_2_O)) simulated UV–vis spectra for TAMRA, calculated with a varying number of excited states.

Since the two lowest‐energy excited states account for the longest‐wavelength part of the spectrum in the case of B3LYP, we investigated whether higher‐energy states influence this spectral range. To test this, calculations involving 3 and 20 excitations were performed. The results are presented in Figure 5. We show that reproducing the spectral features above 450 nm requires including only the three lowest‐lying excited states, which should significantly reduce the computational cost for TAMRA‐labeled peptides.

Based on the series of tests, B3LYP with the 6‐31+G(d) basis set, without dispersion correction, and involving only the three lowest‐lying excited states, provides an optimal balance of computational efficiency and accuracy in reproducing the spectral characteristics of TAMRA. Larger basis sets and dispersion corrections, as well as including more excited states, did not improve the results. Therefore, this computational protocol will be used in further studies.

Given that the experimental spectra were measured in water, the impact of explicit solvent molecules on the UV–vis spectrum was investigated. To this end, 10 clusters consisting of the fluorophore and four water molecules each were prepared. These clusters were selected from snapshots of MD simulations where the water molecules were in close proximity to the amine groups of TAMRA. Geometry optimizations of each cluster resulted in structures consistent with those obtained from continuum solvent calculations, specifically featuring a planar 3‐*N*,3‐*N*,6‐*N*,6‐*N*‐tetramethyl‐9*H*‐xanthene‐3,6‐diamine moiety. In the studied clusters, although the water molecules engage in hydrogen bonding with the carboxylate groups and form weak contacts with the aromatic xanthene rings (Figure S2), these interactions do not influence the lowest‐energy band of the UV–vis spectrum or the frontier MOs (Figure S3).

### 3.3 UV–Vis Spectra Averaged for Selected MD Snapshots

**FIGURE 6:**
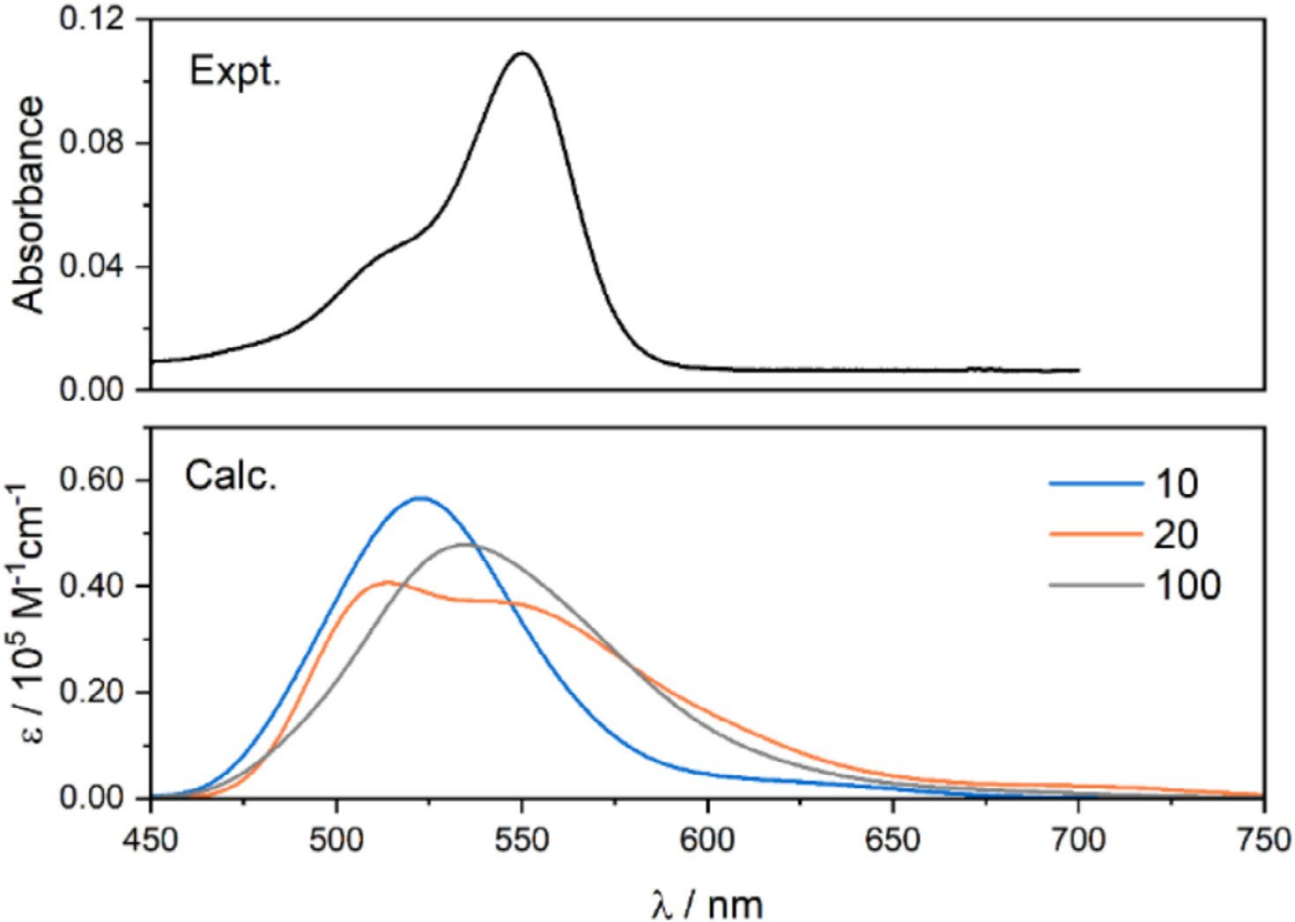
Experimental and simulated (based on B3LYP/6‐31+G(d)/PCM(H_2_O) calculations, averaged over 10, 20, and 100 MD snapshots) UV–vis spectra for TAMRA.

In Figure 6, the simulated UV–vis absorption spectra for TAMRA averaged over 10, 20, and 100 randomly selected snapshots from MD simulation are shown. As expected, the lowest‐energy band broadens compared to the spectrum obtained for the DFT‐optimized structure, and the two bands are not distinctly separated. Additionally, there is some absorption intensity at longer wavelengths than the main absorption peak, indicating that certain conformations exhibit lower‐lying excitations with non‐negligible oscillator strengths. However, the position of the maximum shifts for the spectrum averaged over 10 snapshots is closer to the experimental data and even more so for 20 snapshots. Notably, neither selection of a different set of snapshots nor increasing their number to 100 results in further improvement (Figures 6 and S4).

**FIGURE 7:**
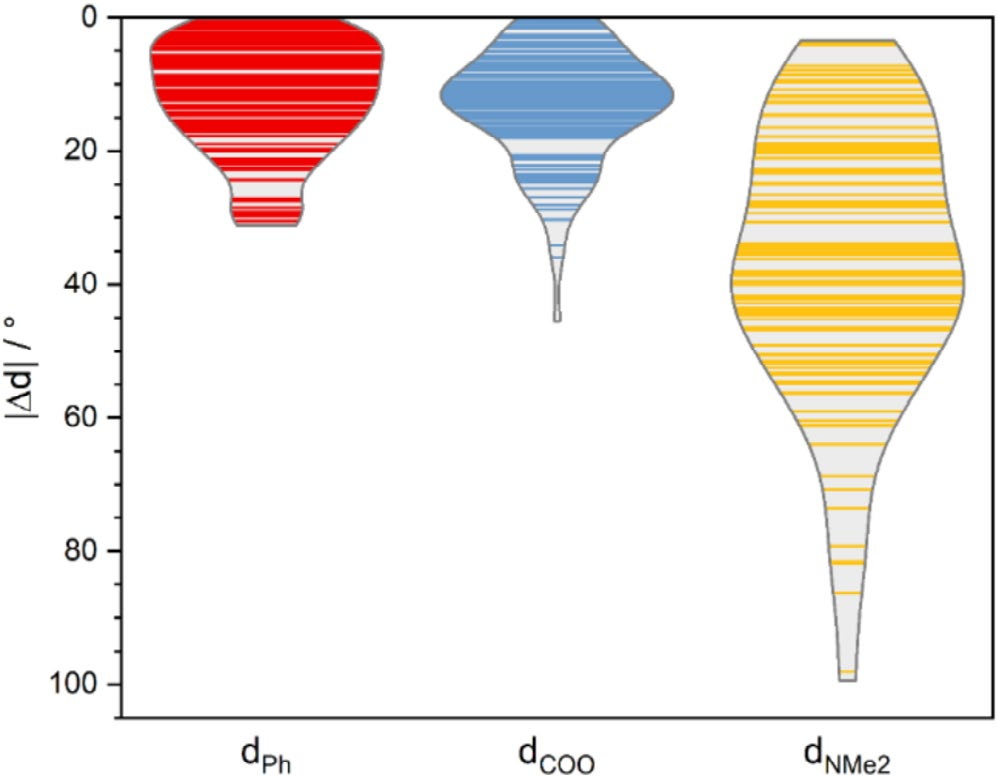
Visualization of deviations in selected dihedral angles of TAMRA from 100 MD snapshots relative to the optimized DFT structure. The violin plots represent data distributions based on kernel density estimation using Scott's rule as implemented in OriginPro 2024b [43].

To elucidate the origins of the absorption response, we analyzed the deviations in dihedral angles between the dicarboxylate phenyl moiety and xanthene, dimethylamine and xanthene, as well as between the carboxylate group and phenyl (see Figure 1 for the definition of dihedral angles) across 100 MD snapshots, comparing them to the values obtained from the optimized B3LYP structure. The results are presented in Figure 7. As shown in Figure 7, for the phenyl group, the majority of conformations are nearly perpendicular to xanthene, similar to the DFT‐optimized structure, with deviations of up to 20°. In the case of the carboxylate group, most conformations exhibit a twist of approximately 10°–15° relative to the phenyl ring. For the dimethylamine moiety, a wider variety of conformations is observed, with deviations from the optimized structure of up to 100°, with a density peak around 40° (see Figure S5 for rotamer populations).

### 3.4 UV–Vis Spectra for Different Conformations of TAMRA

**FIGURE 8:**
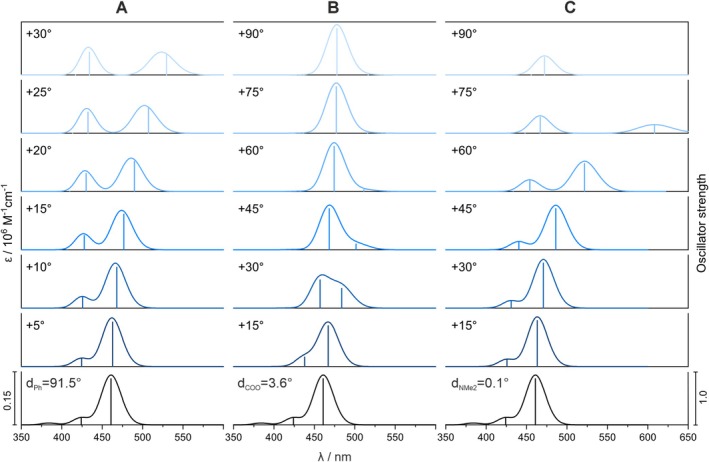
TD‐DFT simulated UV–vis spectra for TAMRA conformations, based on rotations of phenyl (A), carboxyl (B), and dimethylamine (C) groups by specified dihedral angles (see Figure 1 for the definition of dihedral angles). Calculated excitation energies and oscillator strengths are represented as stick spectra. B3LYP/6‐31+G(d)/PCM(H_2_O) calculations.

To further investigate the influence of structural changes on the absorption characteristics of the dye, constrained calculations were performed for the B3LYP‐optimized structure by rotating the phenyl, carboxylate, and amine groups. The results, shown in Figure 8, reveal that these structural variations have a significant impact on the spectral lines.

**FIGURE 9:**
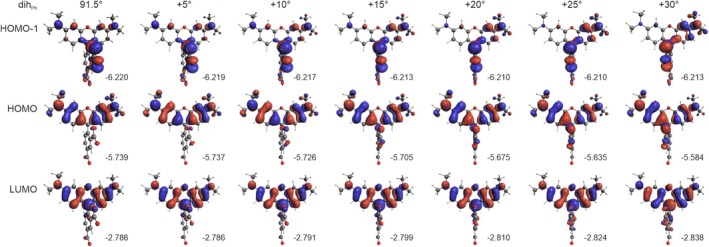
Isosurfaces (± 0.03 au) of MOs involved in dominated transitions for TAMRA conformations, based on rotations of the phenyl group (**A**). The numbers listed are orbital energy values (in eV). B3LYP/6‐31+G(d)/PCM(H_2_O) calculations.

As seen in Figure 8A, the rotation of the dicarboxylate phenyl moiety leads to a gradual exchange of intensity between the two lowest‐energy excitations, accompanied by a noticeable red shift of the lowest‐energy band. While the overall assignment of both bands remains unchanged, there is a decrease in the CT character of the second excitation (Table 4, Figure 9). An inspection of the frontier MOs energies (Figure 9) reveals that the redshift of the first band is primarily due to a reduction in the HOMO‐LUMO gap, driven by increased destabilization of the HOMO and, to a lesser extent, LUMO stabilization.

**TABLE 4:** Dominant excitations and corresponding occupied → unoccupied MO‐pair contributions (> 10%) for TAMRA conformations, based on rotations around phenyl, carboxyl, and dimethylamine groups by a specified dihedral angles (Δ*d*).

Rotated group	Δ*d* [°]	Excit. no.	*E* (eV)	*λ* (nm)	*f*	Occ no.	Unocc no.	%
B3LYP/6‐31+G(d)	0	1	2.69	460	0.8695	113	114	98.6
		2	2.92	424	0.1257	112	114	95.1
Ph	5	1	2.68	462	0.8498	113	114	97.9
		2	2.92	424	0.1456	112	114	94.5
	10	1	2.66	466	0.7848	113	114	96.0
		2	2.92	425	0.2023	112	114	92.6
	15	1	2.62	474	0.6893	113	114	93.5
		2	2.91	427	0.2813	112	114	89.9
	20	1	2.55	486	0.5837	113	114	91.0
		2	2.89	429	0.3631	112	114	87.2
	25	1	2.47	502	0.4839	113	114	89.2
		2	2.88	431	0.4329	112	114	84.9
	30	1	2.37	523	0.4004	113	114	88.2
		2	2.86	433	0.4819	112	114	83.5
						113	114	10.5
COO^−^	15	1	2.65	467	0.7715	113	114	92.7
		2	2.83	438	0.1788	112	114	88.5
	30	1	2.56	484	0.3733	113	114	58.1
						112	114	39.5
		2	2.71	457	0.5327	112	114	57.5
						113	114	40.4
	45	1	2.47	501	0.1101	112	114	73.2
						113	114	25.1
		2	2.65	468	0.7761	113	114	73.2
						112	114	25.5
	60	1	2.43	511	0.0308	112	114	90.1
		2	2.61	474	0.8521	113	114	89.5
	75	1	2.40	516	0.0094	112	114	96.6
		2	2.60	477	0.8769	113	114	95.9
	90	1	2.40	517	0.0072	112	114	97.5
		2	2.59	478	0.8778	113	114	96.7
NMe_2_	15	1	2.68	463	0.8670	113	114	98.6
		2	2.91	426	0.1267	112	114	94.8
	30	1	2.63	471	0.8496	113	114	98.8
		2	2.88	431	0.1306	112	114	93.8
	45	1	2.55	486	0.7784	113	114	98.9
		2	2.81	440	0.1442	112	114	92.1
	60	1	2.38	522	0.5290	113	114	97.1
		2	2.73	454	0.2044	112	114	88.8
	75	1	2.04	608	0.1501	113	114	96.3
		2	2.66	467	0.3026	112	114	84.8
						110	114	10.3
	90	1	1.80	691	0	113	114	98.1
		2	2.63	472	0.3312	112	114	82.8
						110	114	13.2

*Note:* B3LYP/6‐31+G(d)/PCM(H_2_O) calculations.

**FIGURE 10:**
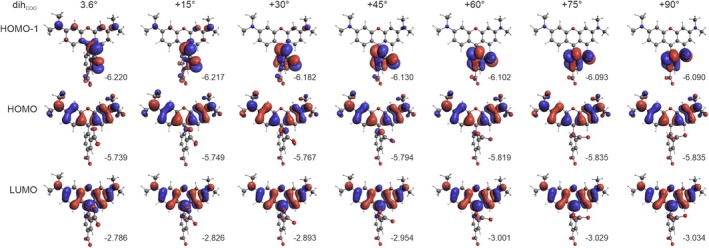
Isosurfaces (± 0.03 au) of MOs involved in dominated transitions for TAMRA conformations, based on rotations of the carboxyl group (**B**). The numbers listed are orbital energy values (in eV). B3LYP/6‐31+G(d)/PCM(H_2_O) calculations.

For the carboxylate group rotation, at a 30° twist, Excitations 1 and 2 are mixed (Figure 8B), showing nearly equal contributions from HOMO‐to‐LUMO and HOMO‐1‐to‐LUMO transitions, with similar intensities (Table 4). As the rotation progresses, the two low‐energy bands switch assignments: Excitation 2 becomes HOMO‐to‐LUMO, maintaining the same position and intensity as Excitation 1 before the exchange, while Excitation 1 shifts to HOMO‐1‐to‐LUMO with a pure CT character (Figure 10), which may explain its diminishing intensity. The red shift of the latter can be attributed to an increase in the HOMO‐1 energy, while the energy of LUMO decreases (as that of HOMO, explaining why the more intense band position remains unchanged).

**FIGURE 11:**
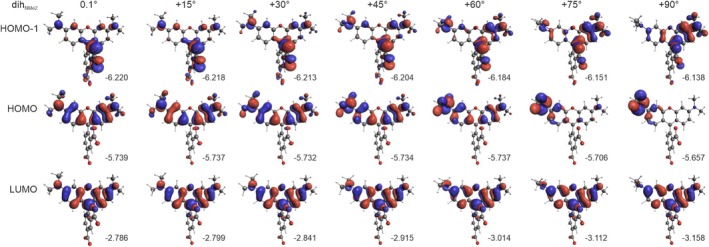
Isosurfaces (± 0.03 au) of MOs involved in dominated transitions for TAMRA conformations, based on rotations of the dimethylamine group (**C**). The numbers listed are orbital energy values (in eV). B3LYP/6‐31+G(d)/PCM(H_2_O) calculations.

In the case of the dimethylamine group rotation, similar behavior is observed as with the phenyl moiety rotation—namely, the exchange of intensities and a redshift of the lowest‐energy band (Figure 8C). When the dimethylamine group is rotated by 90° relative to the xanthene moiety, the lowest‐energy band disappears as the oscillator strength of Excitation 1 drops to zero (Table 4). This is due to a change in the electron density distribution of the HOMO from being delocalized across the entire *π*–electron system to becoming localized on the twisted dimethylamine group (Figure 11). Consequently, Excitation 1 changes from a pure *π*–π* transition to a CT transition. The red shift of the lowest‐energy band, along with the smaller shift of the second band, is primarily due to LUMO stabilization.

The discrepancy between the absorption spectra of DFT‐optimized structures and averaged spectra from MD simulations can be explained by differences in the dimethylamine group orientation. In MD simulations, the most probable angle of this group is shifted by about 40°–60° relative to the optimized DFT structure (Figure 7), resulting in a redshift of nearly 80 nm in the peak position (Figure 8C) and significantly improving agreement with experimental spectra. This shift is a result mainly of LUMO stabilization (from −2.79 eV at planar geometry to −2.92 eV at the angle of 45°) rather than destabilization of HOMO orbitals (from −5.74 to −5.73 eV at the same angles) as seen in Figure 11. In contrast, the phenyl and carboxyl groups (Figure 8A,B) show minimal deviation from the optimized DFT structure, thus having no significant effect on the simulated UV spectra. The calculated rotational barrier for the dimethyl group in TAMRA B3LYP PCM calculations is below 12 kcal/mol and can be practically overcome at room temperature, although the vast majority of the conformations remain planar (Figure S6). Therefore, the rotational flexibility of the dimethylamine group is effectively captured only in classical MD simulations, accounting for the improved match of calculated spectra with experimental data.

**FIGURE 12:**
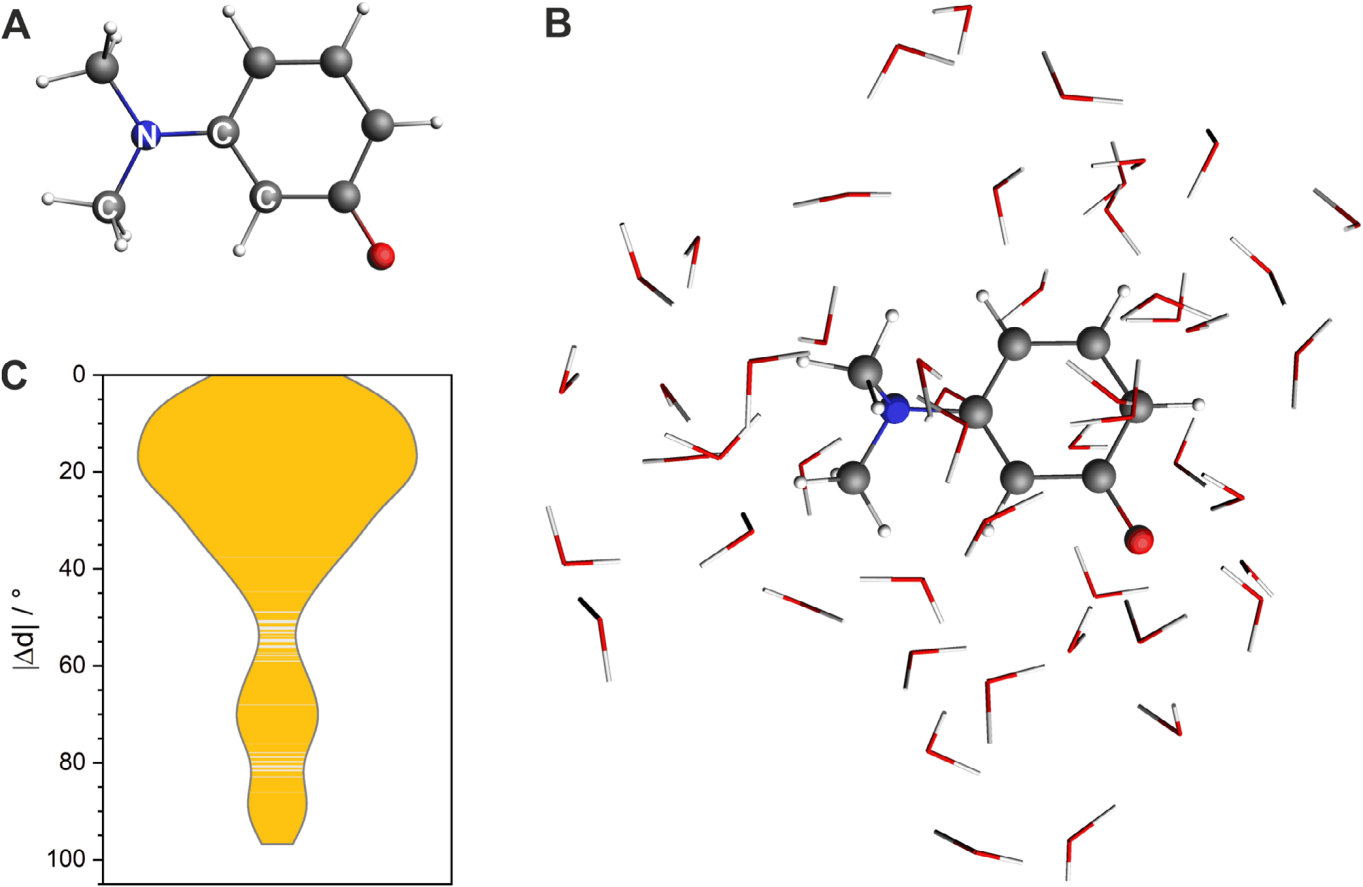
Selected results for the simplified model of TAMRA: (A) the geometry optimized at the B3LYP/6‐31G(d)/PCM(H_2_O) level of theory; (B) a representative snapshot from the Born–Oppenheimer MD trajectory at B3LYP/6‐31G(d); (C) a visualization of deviations from planarity in the C–N–C–C dihedral angle (see (A)) from the MD snapshots. The violin plots represent data distributions based on kernel density estimation using Scott's rule as implemented in OriginPro 2024b [43].

To check whether the dihedral angle of the dimethylamine group is indeed well described in the CGenFF force field used in classical MD simulations with explicit water molecules, we performed BOMD simulations at the B3LYP/6‐31G(d) level of theory with a TAMRA model, 3‐(dimethylamino)phenolate (Figure 12A). The analysis of 20 ps of BOMD simulations revealed that the dihedral angle of the dimethylamine group is not planar, in contrast to PCM simulations without explicit water molecules or with four explicit water molecules, where the dihedral angle of the dimethylamine group was planar. The example of an MD snapshot observed in BOMD simulations is visualized in Figure 12B, whereas the distribution of the dihedral angle during the BOMD simulation of the dimethylamine group is shown in Figure 12C. The results show that the dihedral angle of the dimethylamine group is not planar, and it assumes the most probable value of ca 20°. This is a consequence of explicit water solvation, which results in the reorientation of the dimethylamine group to better interact with neighboring water molecules. Also, this group is quite floppy, assuming values up to 90°. These results further show that classical MD simulations of TAMRA with explicit water molecules capture the flexibility of the dihedral angle of the dimethylamine group quite well. Importantly, the TAMRA model without explicit water in the PCM continuum model resulted in the planar structure (Figure 12A).

Taking BOMD simulation results into account, we show that the force field parameterization of the dihedral angle of the dimethylamine group in classical MD simulations is qualitatively (and quantitatively to some level) correct and is the reason for the existence of non‐planar TAMRA structures in explicit water leading to the experimentally observed red shift in the TAMRA absorption spectrum compared to DFT PCM calculations of TAMRA without explicit solvation.

### 3.5 UV–Vis Spectra for TAMRA With Peptides

**FIGURE 13:**
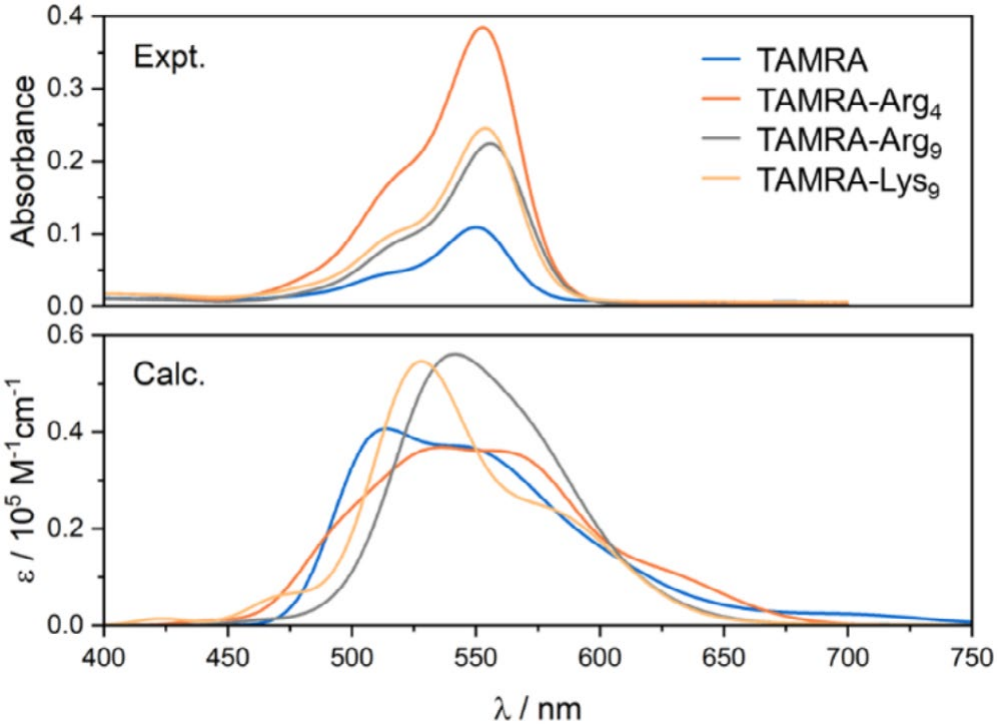
Experimental and simulated (based on B3LYP/6‐31+G(d)/PCM(H_2_O) calculations, averaged over 20 MD snapshots) UV–vis spectra for TAMRA and TAMRA‐labeled peptides (TAMRA‐Arg_4_, TAMRA‐Arg_9_, TAMRA‐Lys_9_).

Figure 13 presents a comparison of the experimental and TD‐DFT simulated UV–vis absorption spectra, averaged over 20 MD snapshots, for both TAMRA and TAMRA‐labeled peptides. As shown in Figure S4, it is not important which set of 20 configurations, out of 100, are taken to reproduce the calculated spectra of TAMRA in water. Therefore, we decided to take 20 snapshots to predict the experimental spectra of different TAMRA‐labeled peptides since the calculations using 100 snapshots are computationally prohibitive, especially for nona‐peptides labeled with TAMRA. The calculated results are in agreement with the experimental results, and the attachment of TAMRA to peptides has no significant effect on the position of the lowest‐energy spectral range, although the calculations do not accurately reproduce the relative intensity. The similarity between the spectra with and without attached peptides arises from the fact that the excitations contributing significant oscillator strength involve *π*–π* transitions within the aromatic region of the dye (Figures 3 and 11), and no further delocalization across the peptide moiety was observed. Importantly, our calculations for labeled peptides agree with the experimental spectra, where no differences in the absorption peaks are observed as well.

**FIGURE 14:**
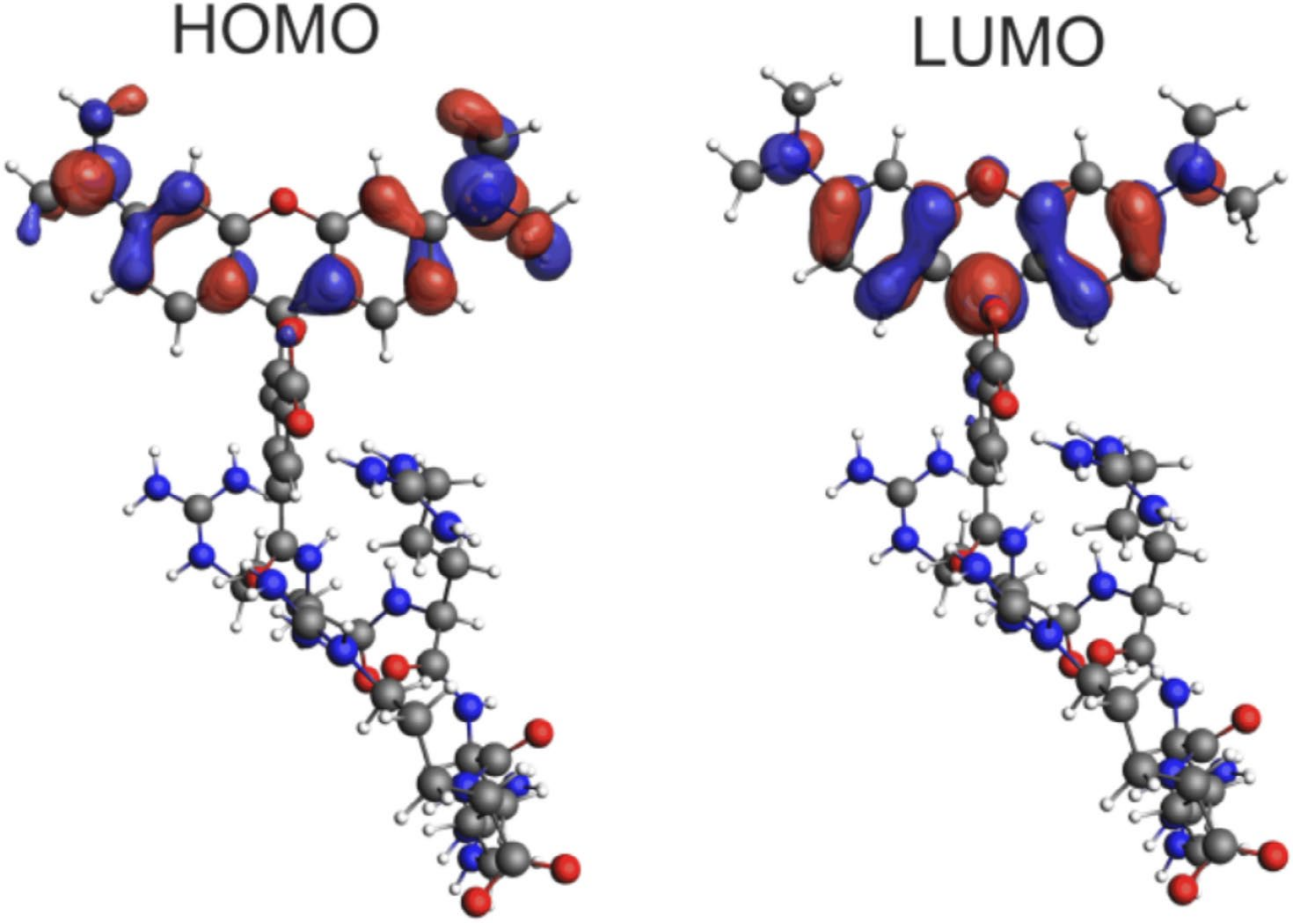
Representative isosurfaces (± 0.03 au) of frontier MOs involved in the dominant transition of TAMRA‐Arg_4_ obtained with B3LYP/6‐31+G(d)/PCM(H_2_O) calculations.

These results are helpful for biophysical studies with TAMRA‐labeled peptides. In particular, TAMRA is often used as a covalently labeled fluorescent probe in fluorescence investigations of cell‐penetrating peptide translocation, where polyarginine is one of the main subjects of interest [44]. It is, therefore, important to know how the UV response of TAMRA‐labeled peptides compares to TAMRA itself. The current results show that the main contribution to the UV absorption spectrum of TAMRA is unchanged upon the addition of the peptide to the carboxyl group of TAMRA, regardless of the peptide structure or size. These results show that TAMRA can be used reliably in peptide labeling, especially with peptides with arginine and lysine residues. In future studies, it will be interesting to find out how other peptides used in fluorescence experiments, such as transmembrane proteins rich in tryptophane residues [45], influence the UV absorption spectra of TAMRA or other fluorescent probes used in the community (Figure 14).

## 4 Conclusions

This study of the UV absorption spectra of TAMRA, using MD simulations and TD‐DFT calculations, provides insights into how structural dynamics impact its spectral properties. Key findings reveal that the rotational flexibility of the dimethylamine group in TAMRA plays a central role in its calculated absorption spectrum. MD simulations showed that the most probable angle of this group varies by 40°–60° from the optimized geometry found with DFT, causing a redshift of nearly 80 nm in the absorption peak, closely matching experimental UV–vis spectra. In contrast, the phenyl and carboxyl groups, with minimal orientation deviation, contribute little to UV absorption shifts, emphasizing the influence of the dimethylamine group in solvated environments. BOMD at the DFT level of theory for the TAMRA model also confirmed that the rotational flexibility of the dimethylamine group in TAMRA occurs due to explicit solvation and interaction of the dimethylamine group with neighboring water molecules.

Additionally, attaching TAMRA to peptides (Arg_4_, Arg_9_, and Lys_9_) does not significantly change the position of the main UV absorption peak, a finding consistent in both simulations and experiments. Peptide conjugation leaves the *π*–π* transitions in the aromatic region of the dye unaffected, preserving fundamental absorption features, including the peak position and overall spectral shape. By achieving a close agreement with experimental spectra through MD simulation snapshots, the study highlights the potential of this approach to predict fluorophore absorption behavior in complex biological systems, guiding the design of optimized probes for diverse research applications.

## Supporting information


**Figure S1.** Isosurfaces (± 0.03 au) of MOs involved in the selected transitions of TAMRA, calculated using various DFT functionals. Calculations were performed with DFT/6‐31+G(d)/PCM(H_2_O).
**Figure S2**. Optimized ground‐state structure of clusters of TAMRA with four water molecules (B3LYP/6‐31+G(d)/PCM(H_2_O)). The closest intermolecular contacts are given, with distances shorter than 3 Å.
**Figure S3**. Left: Simulated averaged UV–vis spectrum of 10 TAMRA/water clusters derived from MD snapshots compared with the simulated spectrum of the optimized isolated TAMRA structure. Right: Isosurfaces (±0.03 au) of MOs involved in the dominant transition of TAMRA and the representative TAMRA/water cluster. Numbers listed are orbital energies (in eV). B3LYP/6‐31+G(d)/PCM(H_2_O)) calculations.
**Figure S4**. Averaged simulated UV–vis spectra for five sets of 20 randomly selected MD snapshots of TAMRA. The averaged spectrum for these five sets, as well as for the 100 MD snapshots (presented in Figure 6), is shown for comparison. B3LYP/6‐31+G(d)/PCM(H_2_O) calculations.
**Figure S5**. Rotamer populations of TAMRA derived from 100 MD snapshots as a function of the NMe_2_ dihedral angle.
**Figure S6**. Results of the relaxed potential energy surface scan for the two‐way rotation of the dimethylamine group in TAMRA, along with the fully optimized structures of the lowest‐energy conformation and the transition state. The sharp energy stabilization at the angles of 130° (forward rotation) and 50° (reverse rotation) is due to the stabilization induced by the pyramidal inversion of the amine group (in a black circle). Values displayed are calculated energy and free energy barriers (in kcal/mol). B3LYP/6‐31+G(d)/PCM(H_2_O) calculations.

## Data Availability

The data that support the findings of this study are available from the corresponding author upon reasonable request.

## References

[1] A. S. Waggoner and L. Stryer , “Fluorescent Probes of Biological Membranes,” Proceedings of the National Academy of Sciences of the United States of America 67, no. 2 (1970): 579–589. 5289011 10.1073/pnas.67.2.579 PMC283246

[2] D. Wu , A. C. Sedgwick , T. Gunnlaugsson , E. U. Akkaya , J. Yoon , and T. D. James , “Fluorescent Chemosensors: The Past, Present and Future,” Chemical Society Reviews 46, no. 23 (2017): 7105–7123. 29019488 10.1039/c7cs00240h

[3] I. D. Johnson , Molecular Probes Handbook: A Guide to Fluorescent Probes and Labeling Technologies (Life Technologies Corporation, 2010).

[4] L. Cwiklik , A. J. A. Aquino , M. Vazdar , et al., “Absorption and Fluorescence of PRODAN in Phospholipid Bilayers: A Combined Quantum Mechanics and Classical Molecular Dynamics Study,” Journal of Physical Chemistry A 115, no. 41 (2011): 11428–11437. 21910413 10.1021/jp205966b

[5] P. Rybczynski and A. Kaczmarek‐Kȩdziera , “BODIPY Dimers: Structure, Interaction, and Absorption Spectrum,” Structural Chemistry 32, no. 3 (2021): 953–965.

[6] C. Obermaier , A. Griebel , and R. Westermeier , “Principles of Protein Labeling Techniques,” Methods in Molecular Biology 2261 (2021): 549–562. 33421014 10.1007/978-1-0716-1186-9_35

[7] M. S. Goncalves , “Fluorescent Labeling of Biomolecules With Organic Probes,” Chemical Reviews 109, no. 1 (2009): 190–212. 19105748 10.1021/cr0783840

[8] D. Renciuk , J. Zhou , L. Beaurepaire , A. Guedin , A. Bourdoncle , and J. L. Mergny , “A FRET‐Based Screening Assay for Nucleic Acid Ligands,” Methods 57, no. 1 (2012): 122–128. 22465278 10.1016/j.ymeth.2012.03.020

[9] K. Kawai , E. Matsutani , A. Maruyama , and T. Majima , “Probing the Charge‐Transfer Dynamics in DNA at the Single‐Molecule Level,” Journal of the American Chemical Society 133, no. 39 (2011): 15568–15577. 21875061 10.1021/ja206325m

[10] A. V. Marenich , C. J. Cramer , and D. G. Truhlar , “Universal Solvation Model Based on Solute Electron Density and on a Continuum Model of the Solvent Defined by the Bulk Dielectric Constant and Atomic Surface Tensions,” Journal of Physical Chemistry B 113, no. 18 (2009): 6378–6396. 19366259 10.1021/jp810292n

[11] J. Tomasi , B. Mennucci , and R. Cammi , “Quantum Mechanical Continuum Solvation Models,” Chemical Reviews 105, no. 8 (2005): 2999–3094. 16092826 10.1021/cr9904009

[12] M. J. Frisch , G. W. Trucks , H. B. Schlegel , et al., Gaussian 16, Revision B.01 (Gaussian, Inc, 2016).

[13] A. D. Becke , “Density‐Functional Exchange‐Energy Approximation With Correct Asymptotic Behavior,” Physical Review A 38, no. 6 (1988): 3098–3100. 10.1103/physreva.38.3098 9900728

[14] J. P. Perdew , “Density‐Functional Approximation for the Correlation Energy of the Inhomogeneous Electron Gas,” Physical Review B: Condensed Matter 33, no. 12 (1986): 8822–8824. 9938299 10.1103/physrevb.33.8822

[15] C. Lee , W. Yang , and R. G. Parr , “Development of the Colle‐Salvetti Correlation‐Energy Formula Into a Functional of the Electron Density,” Physical Review B 37, no. 2 (1988): 785–789. 10.1103/physrevb.37.785 9944570

[16] J. Tao , J. P. Perdew , V. N. Staroverov , and G. E. Scuseria , “Climbing the Density Functional Ladder: Nonempirical Meta–Generalized Gradient Approximation Designed for Molecules and Solids,” Physical Review Letters 91, no. 14 (2003): 146401. 14611541 10.1103/PhysRevLett.91.146401

[17] Y. Zhao and D. G. Truhlar , “The M06 Suite of Density Functionals for Main Group Thermochemistry, Thermochemical Kinetics, Noncovalent Interactions, Excited States, and Transition Elements: Two New Functionals and Systematic Testing of Four M06‐Class Functionals and 12 Other Functionals,” Theoretical Chemistry Accounts 120, no. 1–3 (2008): 215–241.

[18] V. N. Staroverov , G. E. Scuseria , J. Tao , and J. P. Perdew , “Comparative Assessment of a New Nonempirical Density Functional: Molecules and Hydrogen‐Bonded Complexes,” Journal of Chemical Physics 119, no. 23 (2003): 12129–12137. 10.1063/1.4971853 28010100

[19] C. Adamo and V. Barone , “Toward Reliable Density Functional Methods Without Adjustable Parameters: The PBE0 Model,” Journal of Chemical Physics 110, no. 13 (1999): 6158–6170.

[20] A. D. Becke , “Density‐Functional Thermochemistry. III. The Role of Exact Exchange,” Journal of Chemical Physics 98, no. 7 (1993): 5648–5652.

[21] T. Yanai , D. P. Tew , and N. C. Handy , “A New Hybrid Exchange–Correlation Functional Using the Coulomb‐Attenuating Method (CAM‐B3LYP),” Chemical Physics Letters 393, no. 1–3 (2004): 51–57.

[22] J. D. Chai and M. Head‐Gordon , “Long‐Range Corrected Hybrid Density Functionals With Damped Atom–Atom Dispersion Corrections,” Physical Chemistry Chemical Physics 10, no. 44 (2008): 6615–6620. 18989472 10.1039/b810189b

[23] S. Grimme , S. Ehrlich , and L. Goerigk , “Effect of the Damping Function in Dispersion Corrected Density Functional Theory,” Journal of Computational Chemistry 32, no. 7 (2011): 1456–1465. 21370243 10.1002/jcc.21759

[24] S. Grimme , J. Antony , S. Ehrlich , and H. Krieg , “A Consistent and Accurate ab Initio parametrization of Density Functional Dispersion Correction (DFT‐D) for the 94 Elements H‐Pu,” Journal of Chemical Physics 132, no. 15 (2010): 154104. 20423165 10.1063/1.3382344

[25] R. Krishnan , J. S. Binkley , R. Seeger , and J. A. Pople , “Self‐Consistent Molecular Orbital Methods. XX. A Basis Set for Correlated Wave Functions,” Journal of Chemical Physics 72, no. 1 (1980): 650–654.

[26] M. J. Frisch , J. A. Pople , and J. S. Binkley , “Self‐Consistent Molecular Orbital Methods 25. Supplementary Functions for Gaussian Basis Sets,” Journal of Chemical Physics 80, no. 7 (1984): 3265–3269.

[27] T. H. Dunning , “Gaussian Basis Sets for Use in Correlated Molecular Calculations. I. The Atoms Boron Through Neon and Hydrogen,” Journal of Chemical Physics 90, no. 2 (1989): 1007–1023.

[28] A. K. Wilson , D. E. Woon , K. A. Peterson , and T. H. Dunning , “Gaussian Basis Sets for Use in Correlated Molecular Calculations. IX. The Atoms Gallium Through Krypton,” Journal of Chemical Physics 110, no. 16 (1999): 7667–7676.

[29] R. A. Kendall , T. H. Dunning , and R. J. Harrison , “Electron Affinities of the First‐Row Atoms Revisited. Systematic Basis Sets and Wave Functions,” Journal of Chemical Physics 96, no. 9 (1992): 6796–6806.

[30] M. Cossi and V. Barone , “Time‐Dependent Density Functional Theory for Molecules in Liquid Solutions,” Journal of Chemical Physics 115, no. 10 (2001): 4708–4717.

[31] C. Møller and M. S. Plesset , “Note on an Approximation Treatment for Many‐Electron Systems,” Physical Review 46, no. 7 (1934): 618–622.

[32] F. Weigend , A. Köhn , and C. Hättig , “Efficient Use of the Correlation Consistent Basis Sets in Resolution of the Identity MP2 Calculations,” Journal of Chemical Physics 116, no. 8 (2002): 3175–3183.

[33] F. Neese , “The ORCA Program System,” WIREs Computational Molecular Science 2, no. 1 (2012): 73–78.

[34] K. Vanommeslaeghe , E. Hatcher , C. Acharya , et al., “CHARMM General Force Field: A Force Field for Drug‐Like Molecules Compatible With the CHARMM All‐Atom Additive Biological Force Fields,” Journal of Computational Chemistry 31, no. 4 (2010): 671–690. 19575467 10.1002/jcc.21367 PMC2888302

[35] S. Macchi , R. Nifosi , G. Signore , et al., “Self‐Aggregation Propensity of the Tat Peptide Revealed by UV‐Vis, NMR and MD Analyses,” Physical Chemistry Chemical Physics 19, no. 35 (2017): 23910–23914. 28836633 10.1039/c7cp04320a

[36] M. Jan Akhunzada , B. Chandramouli , N. Bhattacharjee , S. Macchi , F. Cardarelli , and G. Brancato , “The Role of Tat Peptide Self‐Aggregation in Membrane Pore Stabilization: Insights From a Computational Study,” Physical Chemistry Chemical Physics 19, no. 40 (2017): 27603–27610. 28980686 10.1039/c7cp05103d

[37] J. Huang , S. Rauscher , G. Nawrocki , et al., “CHARMM36m: An Improved Force Field for Folded and Intrinsically Disordered Proteins,” Nature Methods 14, no. 1 (2017): 71–73. 27819658 10.1038/nmeth.4067 PMC5199616

[38] W. Jorgensen , J. Chandrasekhar , J. Madura , R. Impey , and M. Klein , “Comparison of Simple Potential Functions for Simulating Liquid Water,” Journal of Chemical Physics 79 (1983): 926–935.

[39] M. J. Abraham , T. Murtola , R. Schulz , et al., “GROMACS: High Performance Molecular Simulations Through Multi‐Level Parallelism From Laptops to Supercomputers,” SoftwareX 1‐2 (2015): 19–25.

[40] G. Bussi , D. Donadio , and M. Parrinello , “Canonical Sampling Through Velocity Rescaling,” Journal of Chemical Physics 126, no. 1 (2007): 014101. 17212484 10.1063/1.2408420

[41] I. S. Ufimtsev and T. J. Martinez , “Quantum Chemistry on Graphical Processing Units. 3. Analytical Energy Gradients, Geometry Optimization, and First Principles Molecular Dynamics,” Journal of Chemical Theory and Computation 5, no. 10 (2009): 2619–2628. 26631777 10.1021/ct9003004

[42] A. V. Titov , I. S. Ufimtsev , N. Luehr , and T. J. Martinez , “Generating Efficient Quantum Chemistry Codes for Novel Architectures,” Journal of Chemical Theory and Computation 9, no. 1 (2013): 213–221. 26589024 10.1021/ct300321a

[43] OriginLab Corporation , OriginPro, Version 2024b (OriginLab Corporation, 2024).

[44] F. Madani , S. Lindberg , Ü. Langel , S. Futaki , and A. Gräslund , “Mechanisms of Cellular Uptake of Cell‐Penetrating Peptides,” Journal of Biophysics 2011, no. 1 (2011): 414729. 21687343 10.1155/2011/414729 PMC3103903

[45] G. C. N. Thakur , A. Uday , and P. Jurkiewicz , “FRET‐GP—A Local Measure of the Impact of Transmembrane Peptide on Lipids,” Langmuir 39, no. 50 (2023): 18390–18402. 38048524 10.1021/acs.langmuir.3c02505

