# Supplementary material for: UV Absorption Spectra of TAMRA and TAMRA Labeled Peptides: A Combined Density Functional Theory and Classical Molecular Dynamics Study
Source: J Comput Chem. 2025 Mar 31;46(8):e70096. doi: 10.1002/jcc.70096 (PMC11957245; doi:10.1002/jcc.70096)
Supplement: Supplementary file 1 — Figure S1. Isosurfaces (± 0.03 au) of MOs involved in the selected transitions of TAMRA, calculated using various DFT functionals. Calculations were performed with DFT/6‐31+G(d)/PCM(H2O). Figure S2. Optimized ground‐state structure of clusters of TAMRA with four water molecules (B3LYP/6‐31+G(d)/PCM(H2O)). The closest intermolecular contacts are given, with distances shorter than 3 Å. Figure S3. Left: Simulated averaged UV–vis spectrum of 10 TAMRA/water clusters derived from MD snapshots compared with the simulated spectrum of the optimized isolated TAMRA structure. Right: Isosurfaces (±0.03 au) of MOs involved in the dominant transition of TAMRA and the representative TAMRA/water cluster. Numbers listed are orbital energies (in eV). B3LYP/6‐31+G(d)/PCM(H2O)) calculations. Figure S4. Averaged simulated UV–vis spectra for five sets of 20 randomly selected MD snapshots of TAMRA. The averaged spectrum for these five sets, as well as for the 100 MD snapshots (presented in Figure 6), is shown for comparison. B3LYP/6‐31+G(d)/PCM(H2O) calculations. Figure S5. Rotamer populations of TAMRA derived from 100 MD snapshots as a function of the NMe2 dihedral angle. Figure S6. Results of the relaxed potential energy surface scan for the two‐way rotation of the dimethylamine group in TAMRA, along with the fully optimized structures of the lowest‐energy conformation and the transition state. The sharp energy stabilization at the angles of 130° (forward rotation) and 50° (reverse rotation) is due to the stabilization induced by the pyramidal inversion of the amine group (in a black circle). Values displayed are calculated energy and free energy barriers (in kcal/mol). B3LYP/6‐31+G(d)/PCM(H2O) calculations. [file JCC-46-0-s001.docx]

**Supporting Information**

**UV Absorption Spectra of TAMRA And TAMRA Labeled Peptides: A Combined Density Functional Theory and Classical Molecular Dynamics Study**

Mercedes Kukulka^1^, Barbara Pem^2^, Katarina Vazdar^3^, Lukasz Cwiklik^3^ and Mario Vazdar^4,^*

^1^Faculty of Chemistry, Jagiellonian University, Gronostajowa 2, 30-387 Krakow, Poland

^2^Division for Organic Chemistry and Biochemistry, Ruđer Bošković Institute, Bijenička 54, 10000 Zagreb, Croatia

^3^J. Heyrovský Institute of Physical Chemistry of the Czech Academy of Sciences, Dolejškova 2155/3, 182 00 Prague, Czech Republic

^4^Department of Mathematics, Informatics and Cybernetics, University of Chemistry and Technology, Technická 5, 16628 Prague, Czech Republic

*Correspondence to: Mario Vazdar (E-mail: [mario.vazdar@vscht.cz](mailto:mario.vazdar@vscht.cz))

Page 2: Figure S1. Molecular orbitals involved in the selected transitions of TAMRA using different DFT functionals.

Page 3: Figure S2. Optimized ground-state structure of clusters of TAMRA with four water molecules.

Page 4. Figure S3. Simulated UV-vis spectra of TAMRA and TAMRA water clusters.

Page 5. Figure S4. Averaged simulated UV-vis spectra for five sets of 20 randomly selected MD snapshots of TAMRA.

Page 6: Figure S5. Rotamer populations of TAMRA derived from 100 MD snapshots as a function of the NMe_2_ dihedral angle.

Page 7. Figure S6. The relaxed potential energy surface scan for the rotation of the dimethylamine group in TAMRA.


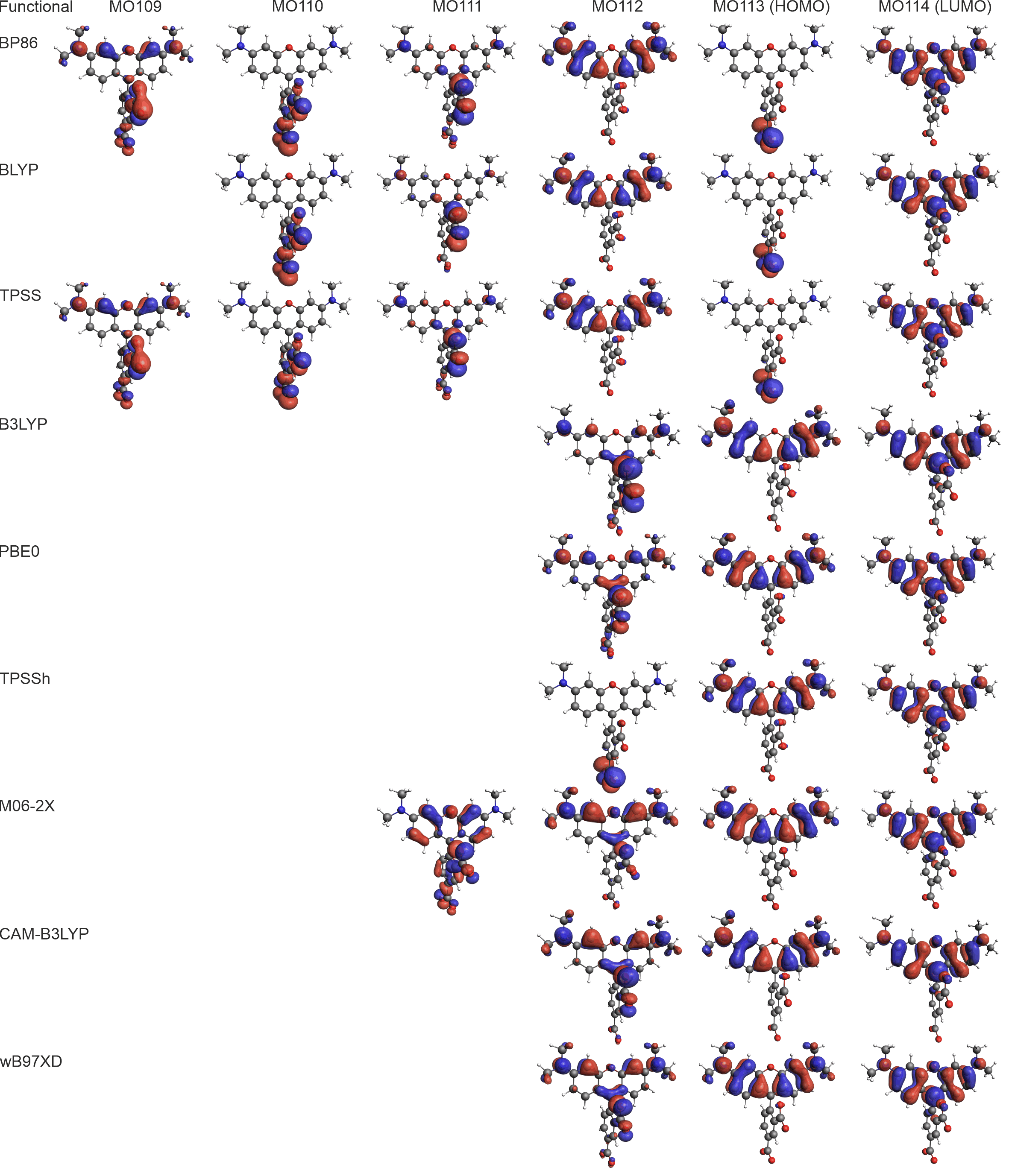
 Figure S1. Isosurfaces (±0.03 au) of MOs involved in the selected transitions of TAMRA, calculated using various DFT functionals. Calculations were performed with DFT/6-31+G(d)/PCM(H_2_O).


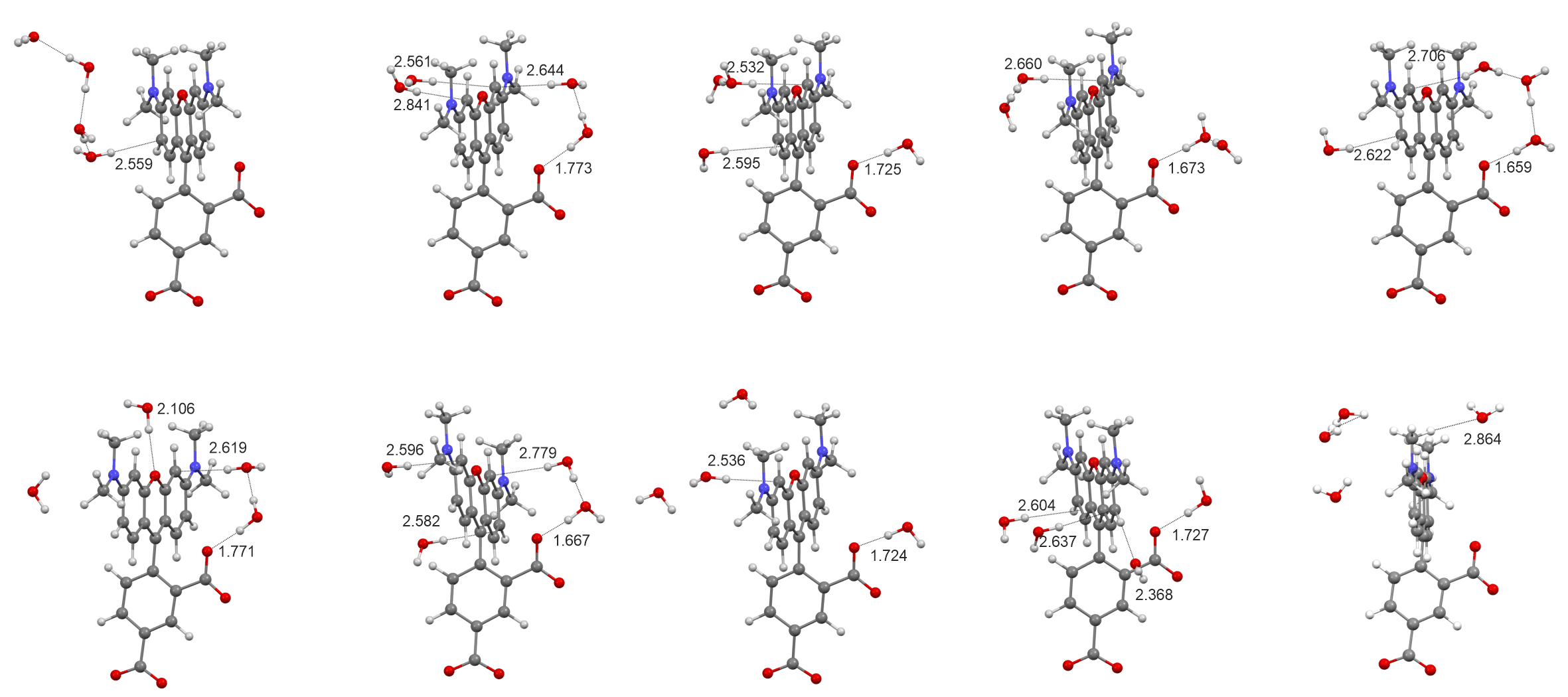


Figure S2. Optimized ground-state structure of clusters of TAMRA with four water molecules (B3LYP/6-31+G(d)/PCM(H_2_O)). The closest intermolecular contacts are given, with distances shorter than 3 Å.


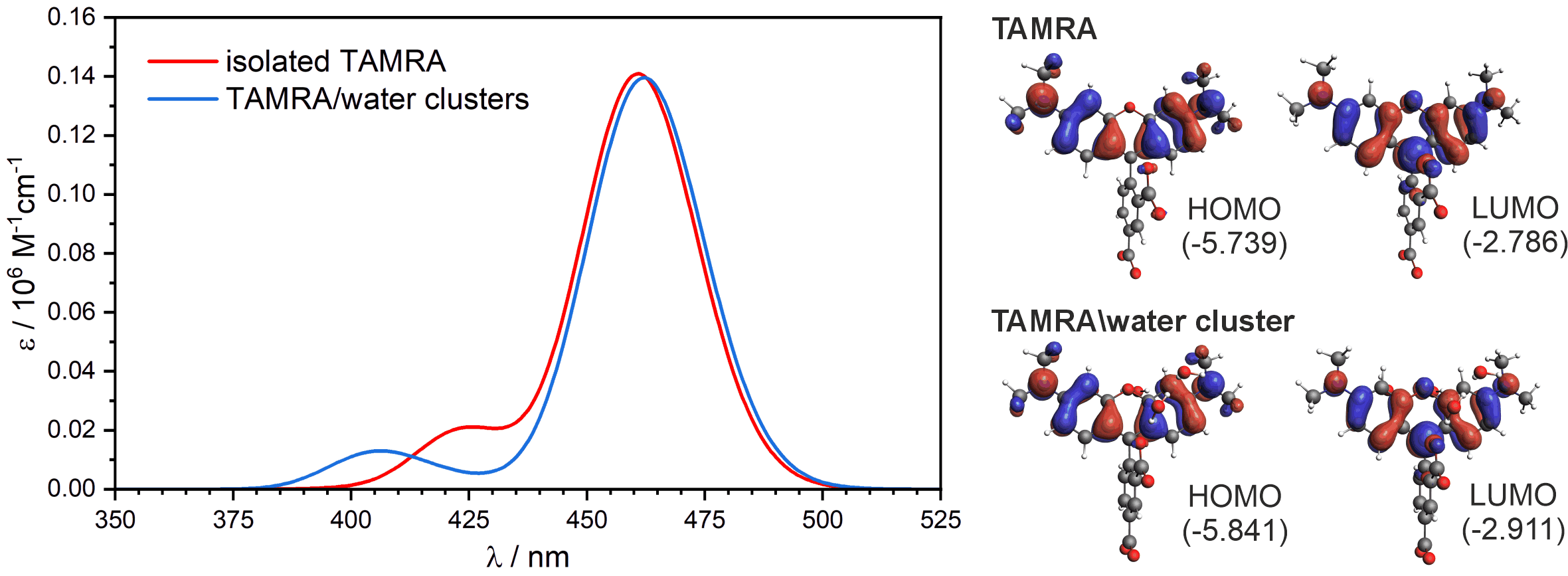


Figure S3. Left: Simulated averaged UV-vis spectrum of ten TAMRA/water clusters derived from MD snapshots compared with the simulated spectrum of the optimized isolated TAMRA structure. Right: Isosurfaces (±0.03 au) of MOs involved in the dominant transition of TAMRA and the representative TAMRA/water cluster. Numbers listed are orbital energies (in eV). B3LYP/6-31+G(d)/PCM(H_2_O)) calculations.


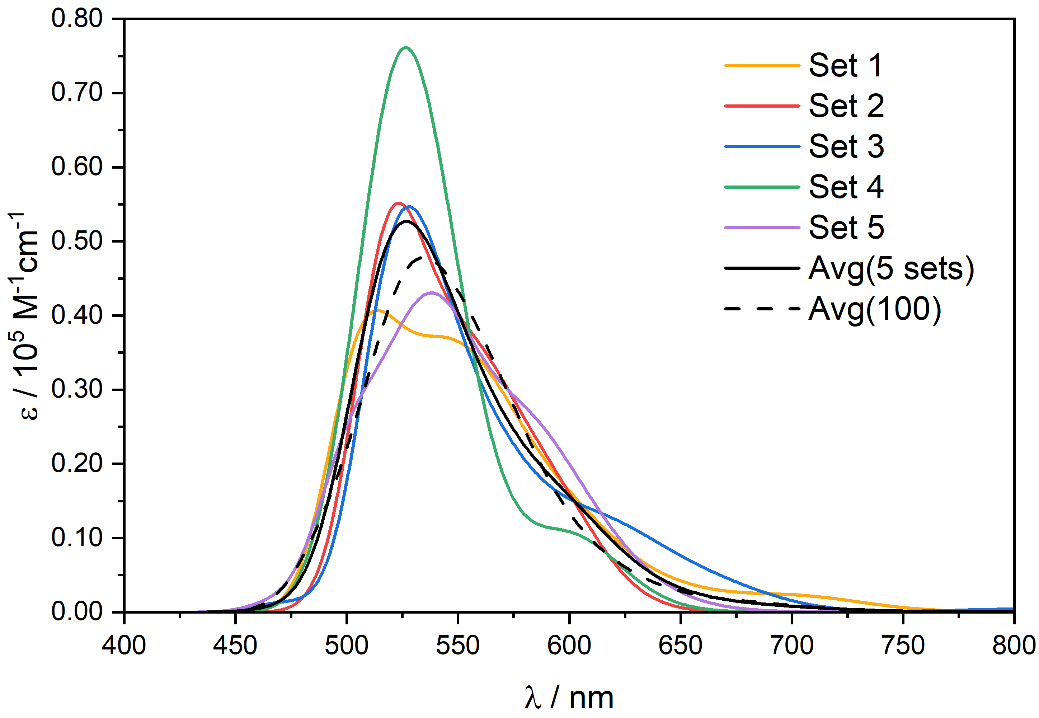


Figure S4. Averaged simulated UV-vis spectra for five sets of 20 randomly selected MD snapshots of TAMRA. The averaged spectrum for these five sets, as well as for the 100 MD snapshots (presented in Figure 6), is shown for comparison. B3LYP/6-31+G(d)/PCM(H_2_O) calculations.


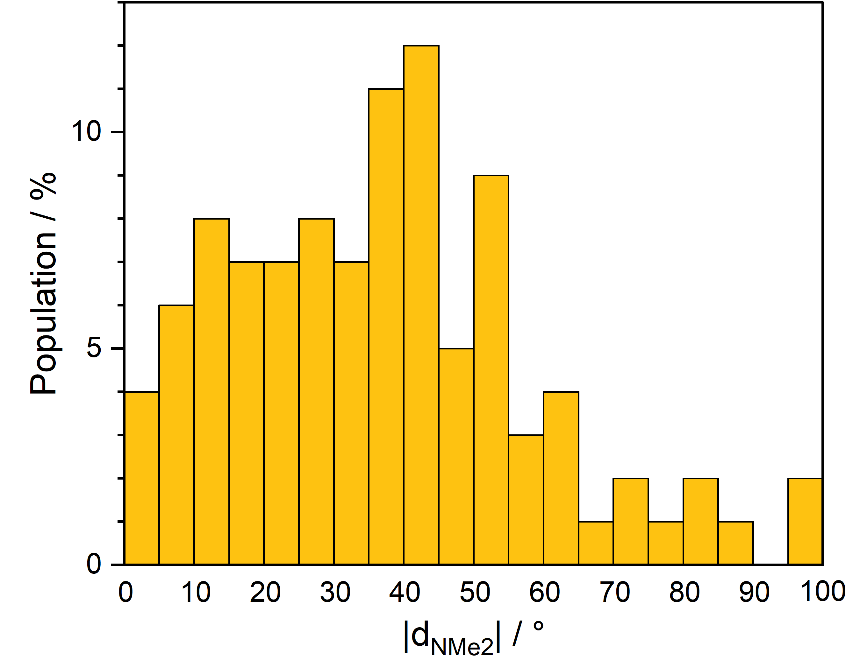


Figure S5. Rotamer populations of TAMRA derived from 100 MD snapshots as a function of the NMe_2_ dihedral angle.


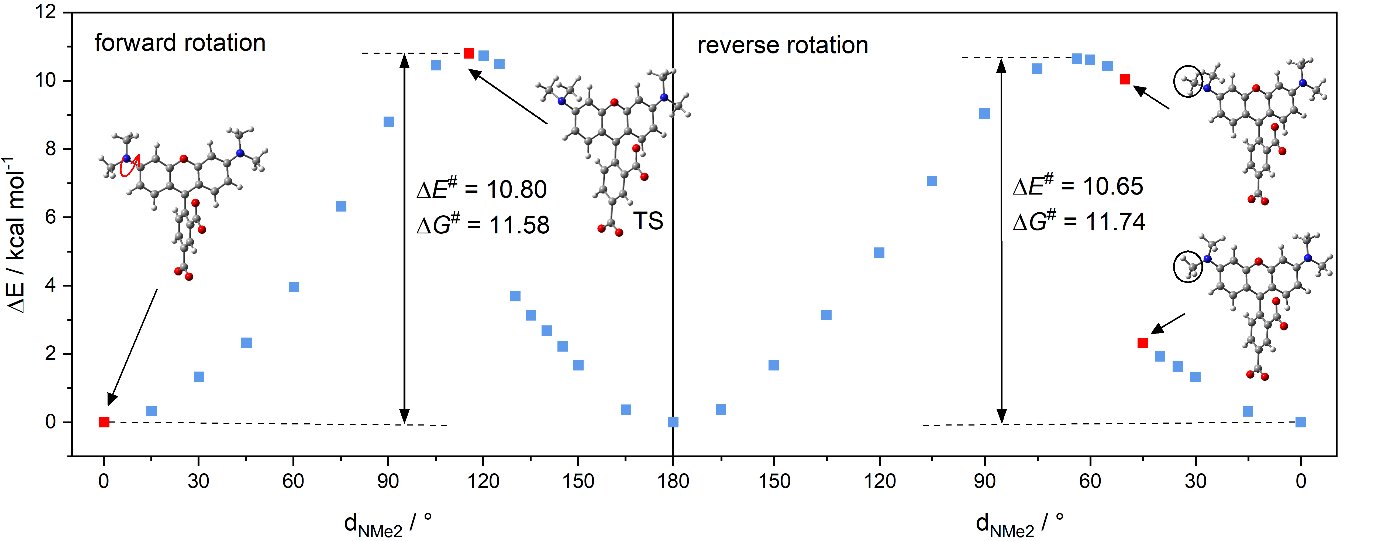


Figure S6. Results of the relaxed potential energy surface scan for the two-way rotation of the dimethylamine group in TAMRA, along with the fully optimized structures of the lowest-energy conformation and the transition state. The sharp energy stabilization at the angles of 130° (forward rotation) and 50° (reverse rotation) is due to the stabilization induced by the pyramidal inversion of the amine group (in a black circle). Values displayed are calculated energy and free energy barriers (in kcal/mol). B3LYP/6-31+G(d)/PCM(H_2_O) calculations.
